# Lower serum IgA is associated with COPD exacerbation risk in SPIROMICS

**DOI:** 10.1371/journal.pone.0194924

**Published:** 2018-04-12

**Authors:** Nirupama Putcha, Gabriel G. Paul, Antoine Azar, Robert A. Wise, Wanda K. O’Neal, Mark T. Dransfield, Prescott G. Woodruff, Jeffrey L. Curtis, Alejandro P. Comellas, M. Bradley Drummond, Allison A. Lambert, Laura M. Paulin, Ashraf Fawzy, Richard E. Kanner, Robert Paine, MeiLan K. Han, Fernando J. Martinez, Russell P. Bowler, R. Graham Barr, Nadia N. Hansel, for the SPIROMICS investigators

**Affiliations:** 1 Johns Hopkins University School of Medicine, Baltimore, Maryland, United States of America; 2 University of North Carolina School of Medicine, Chapel Hill, North Carolina, United States of America; 3 University of Alabama at Birmingham, Birmingham, Alabama, United States of America; 4 University of San Francisco School of Medicine, San Francisco, California, United States of America; 5 University of Michigan Medical School, Ann Arbor, Michigan, United States of America; 6 VA Ann Arbor Healthcare System, Ann Arbor, Michigan, United States of America; 7 University of Iowa College of Medicine, Iowa City, Iowa, United States of America; 8 University of Washington School of Medicine, Seattle, Washington, United States of America; 9 University of Utah Health Sciences Center, Salt Lake City, Utah, United States of America; 10 Department of Veterans Affairs Medical Center, Salt Lake City, Utah, United States of America; 11 University of Utah School of Medicine, Salt Lake City, Utah, United States of America; 12 Weill Cornell Medical College. New York City, New York, United States of America; 13 National Jewish Health, Denver, Colorado, United States of America; 14 Columbia University School of Medicine, New York, New York, United States of America; National and Kapodistrian University of Athens, GREECE

## Abstract

**Background:**

Decreased but measurable serum IgA levels (≤70 mg/dL) have been associated with risk for infections in some populations, but are unstudied in COPD. This study tested the hypothesis that subnormal serum IgA levels would be associated with exacerbation risk in COPD.

**Methods:**

Data were analyzed from 1,049 COPD participants from the observational cohort study SPIROMICS (535 (51%) women; mean age 66.1 (SD 7.8), 338 (32%) current smokers) who had baseline serum IgA measured using the Myriad RBM biomarker discovery platform. Exacerbation data was collected prospectively (mean 944.3 (SD 281.3) days), and adjusted linear, logistic and zero-inflated negative binomial regressions were performed.

**Results:**

Mean IgA was 269.1 mg/dL (SD 150.9). One individual had deficient levels of serum IgA (<7 mg/dL) and 25 (2.4%) had IgA level ≤70 mg/dL. Participants with IgA ≤70 mg/dL were younger (62 vs. 66 years, p = 0.01) but otherwise similar to those with higher IgA. In adjusted models, IgA ≤70 mg/dL was associated with higher exacerbation incidence rates (IRR 1.71, 95% CI 1.01–2.87, p = 0.044) and greater risk for any severe exacerbation (OR 2.99, 95% CI 1.30–6.94, p = 0.010). In adjusted models among those in the lowest decile (<120 mg/dL), each 10 mg/dL decrement in IgA (analyzed continuously) was associated with more exacerbations during follow-up (β 0.24, 95% CI 0.017–0.46, p = 0.035).

**Conclusions:**

Subnormal serum IgA levels were associated with increased risk for acute exacerbations, supporting mildly impaired IgA levels as a contributing factor in COPD morbidity. Additionally, a dose-response relationship between lower serum IgA and number of exacerbations was found among individuals with serum IgA in the lowest decile, further supporting the link between serum IgA and exacerbation risk. Future COPD studies should more comprehensively characterize immune status to define the clinical relevance of these findings and their potential for therapeutic correction.

## Introduction

Chronic obstructive pulmonary disease (COPD) is the third leading cause of death in the U.S. and is associated with increased risk of respiratory infections. Immunoglobulins are known for their critical role in immune response. Selective IgA deficiency, defined as level of serum IgA (<7 mg/dL) accompanied by normal levels of other immunoglobulins, is the most common primary immunodeficiency.[1] Selective IgA deficiency has been associated with higher risk for respiratory infections in the general population.[2] Subnormal serum IgA levels (IgA serum level of ≤70 mg/dL) have also been associated with higher risk of infection in the general population. [3,4] Although the immune function of selective IgA deficiency and subnormal serum IgA levels have been studied in other diseases, such as celiac disease and lymphocytic leukemia,[4] [5] an association with COPD morbidity has not been established.

Despite the lack of existing evidence confirming a role of serum levels of IgA and COPD exacerbations, several studies suggest that there may be a link. Prophylactic antibiotic treatment was recently shown to reduce the number of exacerbations in COPD patients with selective IgA deficiency,[6] showing reduction in exacerbation events among individuals with COPD being treated for immunoglobulin deficiency. Additionally, all previous study populations had either undefined hypogammaglobulinemia [6] or had a low IgA as part of a defined primary immunodeficiency [3, 4]. However the implications of low or low normal levels of serum IgA (levels above the clinical definition for deficiency, 7 mg/dL, but on the lower end of the spectrum of normal) in a general population with COPD is not known.

The goal of this work was to test the hypothesis that subnormal serum IgA levels would be associated with increased risk of future exacerbations in COPD, examining participants from the large, well-characterized Subpopulations and Intermediate Outcome Measures in COPD Study (SPIROMICS).

## Materials and methods

SPIROMICS is a multicenter study of current and former smokers (>20 pack-years) age 40–80, with and without airflow obstruction, as well as healthy, lifelong non-smokers. [7] This analysis includes all current and former smokers with COPD (defined as post-bronchodilator FEV_1_/FVC<0.7) having available data on serum IgA.

### IgA level

Serum IgA (mg/dL) levels were collected at enrollment[8] and available on the first 1,049 of 1,530 COPD participants, measured in two separate batches using the Myriad RBM biomarker discovery platform (Myriad-RBM Inc., Austin TX). Data on IgG levels were unavailable, therefore prevalence of selective IgA deficiency could not be determined. Deficient levels of serum IgA were defined as levels <7mg/dL and subnormal level as ≤70mg/dL (inclusive of those <7) as in previous literature.[9]

### Outcomes

Primary outcome was risk of exacerbations assessed prospectively over follow-up (up to 3 years at yearly clinic visits and quarterly telephone calls). Exacerbations were defined as respiratory events treated with antibiotics and/or steroids, while severe exacerbations required hospitalization or emergency room visits, as described previously.[10]

### Statistical methods

Histograms and descriptive statistics were used to evaluate IgA distribution. Because of the low prevalence of deficient IgA (n = 1), further analyses were limited to the evaluation of subnormal serum IgA, inclusive of the individual with deficiency. T-tests and chi-squared tests were utilized to describe differences in participants based upon IgA category (≤70mg/dL vs. >70mg/dL). Two models were utilized to understand risk for exacerbations associated with IgA: (1) logistic regression, with adjusted odds ratios representing the association of subnormal serum IgA (independent variable) with exacerbation risk (dependent variable); and (2) zero-inflated negative binomial regression models, with incidence rate ratio representing the association of IgA category with incidence rate of exacerbations, relevant given the high prevalence of zero values for exacerbation events and also important given this type of model takes into account multiple events per individual if present. The adjusted continuous association of serum IgA (in 10 mg/dL increments) on number of exacerbations over follow-up in a subgroup with the lowest decile of IgA (<120 mg/dL) was also used to investigate possible dose effect of low levels of IgA with exacerbation risk. It was hypothesized that the full spectrum of serum IgA may not be relevant with regards to exacerbation risk, but that if present, a dose response association may be most relevant to explore in those with IgA in the lowest decile. Models were adjusted for age, gender, race (African-American vs. other), baseline post-bronchodilator FEV_1_ percent predicted, smoking status (current vs. former), batch number (for biomarker run, indicating batch 1 or batch 2), and follow-up time (as a covariate except in negative binomial models where it was specified in modeling).

All analyses were conducted with Stata 12 (Stata Statistical Software: Release 12 (program). College Station, Texas: StataCorp., 2011). P-value <0.05 was the significance threshold for main analysis and <0.10 for interaction terms.[11] Institutional review boards at each center approved SPIROMICS, and all participants provided written informed consent (ClinicalTrials.gov: NCT01969344).

## Results

**Fig 1:**
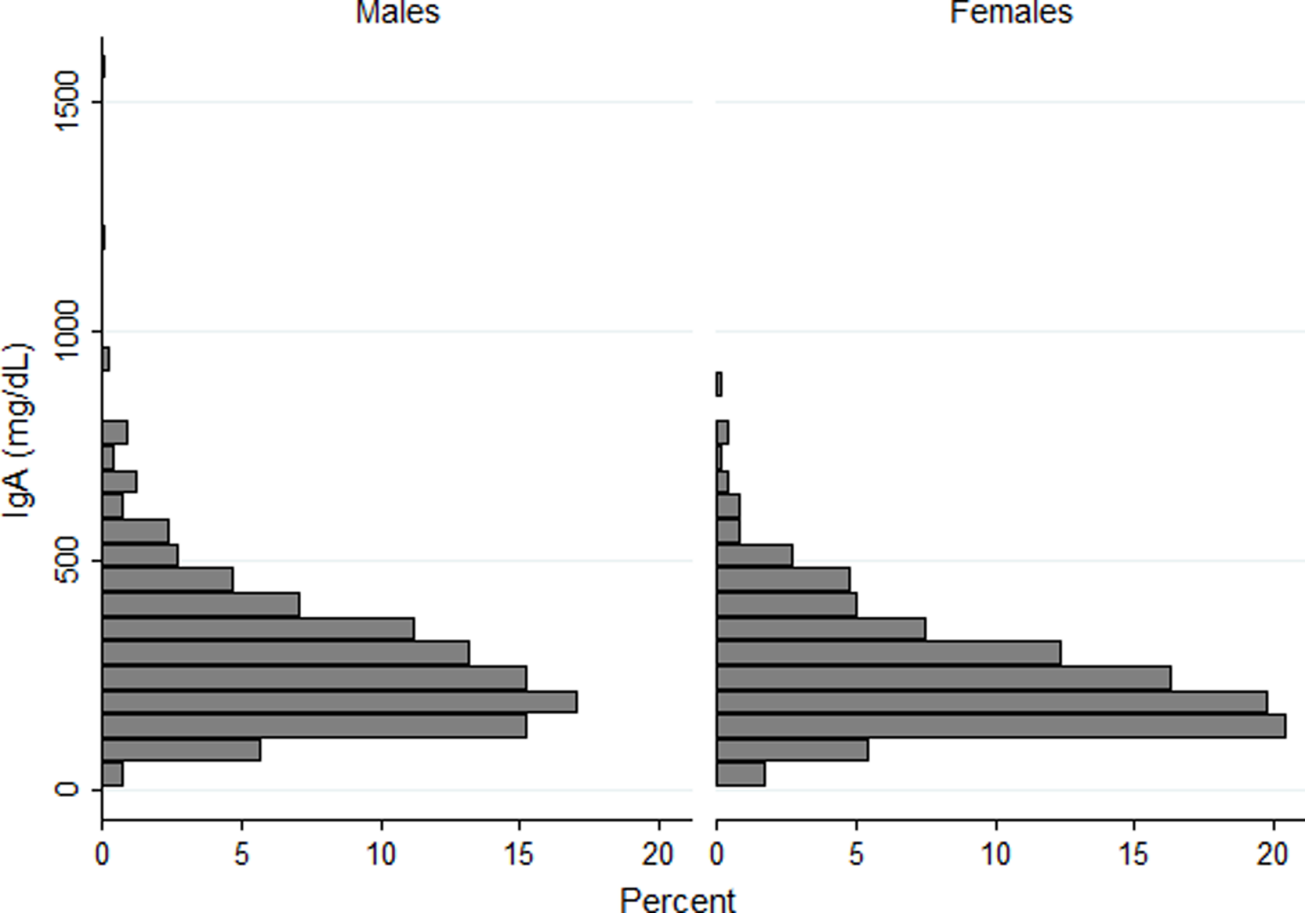
Distribution of serum IgA by gender.

Among the 1,049 COPD participants studied (**Table 1**), the mean IgA level was 269.1 mg/dL (SD 150.9), with median of 240 mg/dL (25^th^ percentile, 75^th^ percentile 170, 340 mg/dL), comparable to levels reported in the general population.[12] One individual had deficient levels of IgA (<7mg/dL), and 25 participants (2.4%) had subnormal serum IgA levels ≤70 mg/dL. Those with subnormal serum IgA were younger (62 vs. 66 years, p = 0.01) and had slightly higher prevalence of women (60% vs. 47%, p = 0.06) but were otherwise similar to those with normal serum IgA (**Table 1**). Distribution of serum IgA by gender in those with COPD is displayed in **Fig 1**.

**Table 1:** Characteristics of COPD participants by serum IgA level ≤70 mg/dL.

Characteristics*	IgA≤70 (n = 25)	IgA>70 (n = 1024)	P-value
**IgA level**	51.2 (16.3)	274 (149)	
**Age**	62.2 (10.07)	66.2 (7.74)	**0.01**
**Female, n(%)**	15 (60%)	420 (41%)	0.06
**African American, n(%)**	2 (8%)	132 (13%)	0.47
**BMI**	27.0 (4.23)	27.5 (5.24)	0.61
**> HS education, n(%)**	16 (64%)	646 (63%)	0.94
**Follow-up time (days)**	1025 (244)	942 (282)	0.15
**Smoking History (Pack-yrs)**	50.5 (24.1)	54.7 (26.7)	0.45
**Current Smokers, n(%)**	12 (48%)	326 (32%)	0.09
**Post-FEV1% Predicted**	63.2 (25.2)	62.3 (23.1)	0.85
**Gold Stage n(%)**			0.71
**1**	8 (32%)	249 (24%)	
**2**	9 (36%)	451 (44%)	
**3**	5 (20%)	236 (23%)	
**4**	3 (12%)	87 (9%)	
**MMRC Dyspnea Score**	1.32 (1.11)	1.21 (1.01)	0.60
**Current oral steroid use, n(%)**	1 (4%)	33 (3%)	0.81
**SGRQ Score**	42.6 (15.5)	35.9 (19.5)	0.09
**Experienced exacerbation over follow-up, n(%)**	15 (60%)	517 (51%)	0.350
**Experienced severe exacerbation over follow-up, n(%)**	12 (48%)	227 (22%)	0.003
**Number of exacerbations per years of follow-up**	1.02 (1.42)	0.584 (0.99)	0.0325
**Number of severe exacerbations per years of follow-up**	0.19 (0.51)	0.24 (0.30)	0.6224
**≥2 exacerbations over follow-up, n(%)**	12 (48%)	309 (31%)	0.06

*All values mean (SD) unless otherwise indicated.

### Association of low IgA levels and exacerbations

Mean number of exacerbations over follow-up was 1.5 (SD 2.6), and 50% experienced any exacerbation event over follow-up (with mean severe exacerbations 0.48, SD 1.3 and 23% experiencing one or more over follow-up). Over a median follow-up time of 2.59 years (25^th^ percentile, 75^th^ percentile 2.08, 3.07 years), participants with subnormal serum IgA tended to have a higher prevalence of ≥2 exacerbations over follow-up compared to those with normal serum IgA (48% vs. 31%; p = 0.06). Subnormal serum IgA was not associated with frequent exacerbator status (2 or more exacerbations) reported over the previous year (16% in those with subnormal serum IgA, 12% without, p = 0.514).

Using adjusted logistic regression, subnormal serum IgA was strongly associated with risk of severe exacerbation (OR 3.37, 95% CI 1.44, 7.88, p = 0.005), although not with an increased risk of any exacerbation (OR 1.26, 95% CI 0.52, 3.04, p = 0.6) **(****Table** 2**)**. When analyzed in a zero-inflated negative binomial regression model, subnormal serum IgA was associated with a 71% higher risk of exacerbations, compared to the group with normal serum IgA (IRR 1.71, 95% CI 1.02–2.88, p = 0.04) **(****Table** 2**)**. IRR for severe exacerbations was not significant (IRR 1.14, 95% CI 0.50 to 2.58, p = 0.8). Sensitivity analysis performed excluding the one individual with levels of IgA consistent with deficiency did not change these findings.

**Table 2:** Association of subnormal serum IgA (<70 mg/dL) with exacerbations.

	Unadjusted analysis			Adjusted analysis*		
	Effect size	95% CI	p-value	Effect size	95% CI	p-value
**Dichotomous Exacerbations (Logistic regression, OR)**	1.47	(0.65, 3.30)	0.353	1.26	(0.52, 3.04)	0.614
**Dichotomous severe exacerbations (Logistic regression, OR)**	**3.20**	**(1.44, 7.10)**	**0.004**	**3.37**	**(1.44, 7.88)**	**0.005**
**Neg binomial exacerbations (IRR)**	1.80	(0.89, 3.64)	0.099	**1.71**	**(1.02, 2.88)**	**0.043**
**Neg binomial severe exacerbations (IRR)**	0.66	(0.31, 1.42)	0.288	1.14	(0.50, 2.58)	0.757

*Adjusted for age, gender, race, baseline FEV1 percent predicted, current smoking status, batch, follow-up time.

### Dose response between serum IgA levels and exacerbations

**Fig 2:**
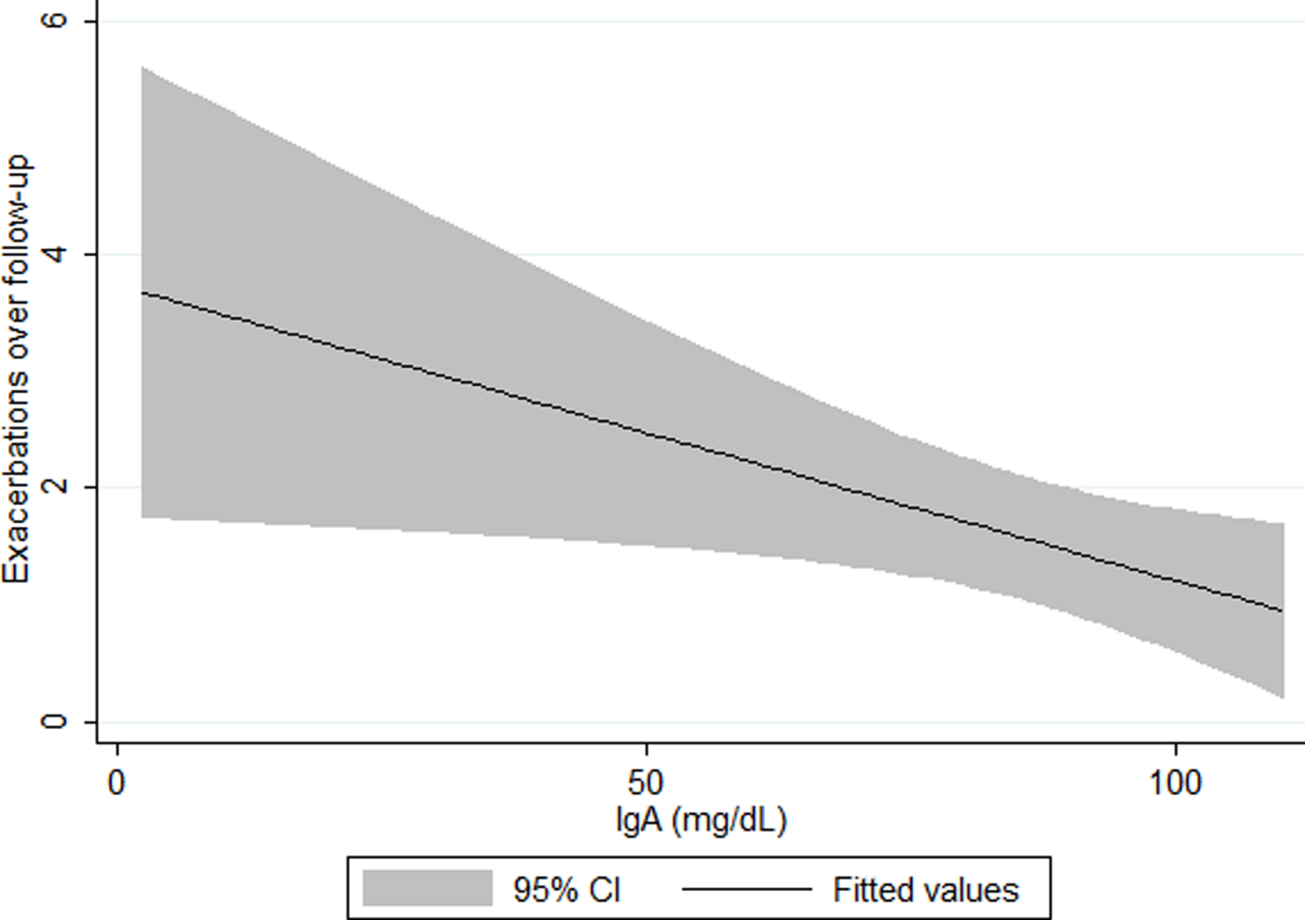
Unadjusted association of IgA with follow-up exacerbations among lowest decile IgA(0–120 mg/dL).

Among the subgroup in the lowest decile of IgA serum levels (<120mg/dL) lower IgA levels were significantly associated in adjusted models, in a dose-response fashion, with higher number of exacerbations **(****Fig** 2**)**; each 10mg/dL decrement in serum IgA was associated with 0.24 more exacerbations over follow-up (β 0.24, 95% CI 0.023, 0.47, p = 0.03). The use of a cutoff of near the 5^th^ percentile (100mg/dL) did not change these observations. Introduction of a quadratic term into this model did not result in a statistically significant coefficient nor a significantly higher likelihood ratio when testing nested models, supporting the assumption of linearity of IgA among those in the lowest decile. Characteristics of participants having IgA level ≤120 and >120 did not differ significantly (see S1 Table in online “Supporting Information”).

## Discussion

In this analysis, a novel association was found between subnormal serum IgA levels (≤70 mg/dL) and prospective risk for COPD exacerbations. Supporting this finding, a significant negative dose-response association between serum IgA levels and burden of exacerbations was identified among individuals in the lowest decile of IgA.

This novel finding of an association of subnormal serum IgA and increased risk for exacerbations in a population with COPD possibly represents an enhanced vulnerability to infection leading to exacerbations. Though low normal levels of serum IgA have been correlated to infection risk in other populations,[2–4] these findings are distinct in that they show risk for disease-specific COPD exacerbation events. This vulnerability to infection is relevant given that bacterial and viral infections are two of the main contributors to COPD exacerbations,[13] accounting for up to an estimated 80% of acute exacerbations.[6]

Previous studies that have investigated the relationship between alterations in serum immunoglobulin levels and COPD mostly focused on immunoglobulins other than IgA, such as IgG.[14,15] COPD patients with depressed IgG and IgG1 serum levels were found to have an increased risk of COPD exacerbations;[14] and patients who had been treated with immunoglobulin therapy and experienced an increase in serum IgG levels had a reduction in the frequency of COPD exacerbations.[15] However, other than case reports,[16] only one study specifically examined serum IgA and exacerbation risk in patients with COPD. This study found that the use of prophylactic antibiotics in those with selective IgA deficiency and COPD was associated with a significant decrease in exacerbations.[6] Notably, the comprehensive biomarker analysis from both COPDGene and SPIROMICS analyzing all biomarkers (n = 90) on the multiplex platform did not find an association of serum IgA with exacerbation risk;[10] however IgA concentrations were modeled continuously and linearly. Overall, the range of serum IgA levels in this population with COPD appears to be comparable to levels reported in the general population[12] and the majority of individuals (~98%) have IgA levels above 70. Thus, the previous biomarker analyses were unable to detect the association of subnormal levels of IgA on exacerbation risk because the negative dose-response relationship between IgA and burden of exacerbations is likely limited to the lower range of IgA values, as detected in this study. The dose response relationship between IgA and exacerbation burden is further supportive of the hypothesis that immune function may play a role in defining exacerbation risk in individuals with COPD and highlights that there could also be a spectrum of increasing risk related to progressively lower IgA levels.

Several studies have also investigated the role of mucosal secretory IgA (SIgA) and COPD severity.[5,17–19] Abnormalities in the epithelial morphology have been shown to be associated with diminished SIgA levels through alterations in expression of the polymeric immunoglobulin receptor leading to increased risk for bacterial invasion, chronic inflammation and ultimately fibrotic remodeling,[17,18] particularly within the small airways.[17,19] It is possible that subnormal total IgA serum levels could be indicative of depressed SIgA levels elsewhere throughout the body. However, to date, the association of serum IgA and airway mucosal SIgA is unknown. One study showed a poor association (p = 0.17) between serum IgA and cervical mucosal SIgA, suggesting that the systemic humoral immune response may not be representative of the local humoral immune response.[20] The findings of this study that serum levels of IgA are associated with exacerbation risk suggest the need to better understand the link between serum and airway IgA and COPD morbidity.

Although this analysis provides novel information related to subnormal serum IgA levels and exacerbation risk in COPD, it does have some limitations. First, though the trends in direction and effect size were consistent across models, there was a lack of consistency of statistically significant findings between models. This lack of consistent findings between models should be considered when interpreting the results of this study. However, given the importance of identifying risk factors for exacerbations and the biological plausibility of relatively low immune function in contributing to risk, these results can be compelling preliminary data for future work. Further, due to incomplete immunological phenotyping, it is not possible to assess potential associations between other immunoglobulins (IgG in particular, which is not measured as part of the Myriad-RBM platform) and COPD exacerbation risk. There was also limited information about precipitant or cause of exacerbations in participants, such as viral or bacterial infection. It should also be noted that to date, this cohort has low overall prevalence and incidence of exacerbations and appears to represent a relatively stable outpatient cohort. Accordingly, it is important to ultimately repeat such an analysis in a group having higher risk for exacerbations in order to understand whether ultimately these findings should inform changes in management. A previous case series among frequent exacerbators described such an association(6) but larger cohort studies of such populations are needed. Additionally, the group of individuals having subnormal serum IgA in this cohort was small and may differ in characteristics from other studies and ultimately the general population with COPD, highlighting the importance of studying this topic in a larger population with more comprehensive immunologic phenotyping.

In conclusion, subnormal serum IgA levels, though infrequent and affecting 2.4% of this COPD cohort, were associated with higher risk for exacerbations in COPD. The negative dose-response relationship between IgA levels and exacerbations among those in the lowest decile of IgA supports a causal role between serum IgA and exacerbation risk and underscores that the increased risk is not limited to those with deficient serum IgA levels (IgA<7). Future studies with comprehensive characterizations of immune status in a larger cohort with COPD are needed to better understand and enrich these findings. These findings are novel and should lead into further investigation of the role of subnormal immunoglobulin levels as a key factor contributing to risk for COPD exacerbations.

## Supporting information

S1 Table Comparison of characteristics of individuals with IgA level less than or equal to 120 compared to those with IgA level greater than 120. (DOCX)

## References

[1] Pallav K, Tariq S, Leffler DA, Dennis M, Hansen J, Peer A et al Serum IgA in Celiac Disease: The Unrecognized Importance of Partial IgA Deficiency. Gastroenterology 140: S-439.

[2] Latiff AH, Kerr MA (2007) The clinical significance of immunoglobulin A deficiency. Ann Clin Biochem 44: 131–139. doi: 10.1258/000456307780117993 17362578 10.1258/000456307780117993

[3] Cunningham-Rundles C, Bodian C (1999) Common variable immunodeficiency: clinical and immunological features of 248 patients. Clin Immunol 92: 34–48. doi: 10.1006/clim.1999.4725 10413651 10.1006/clim.1999.4725

[4] Furst DE (2009) Serum immunoglobulins and risk of infection: how low can you go? Semin Arthritis Rheum 39: 18–29. doi: 10.1016/j.semarthrit.2008.05.002 18620738 10.1016/j.semarthrit.2008.05.002

[5] Pilette C, Durham SR, Vaerman JP, Sibille Y (2004) Mucosal immunity in asthma and chronic obstructive pulmonary disease: a role for immunoglobulin A? Proc Am Thorac Soc 1: 125–135. doi: 10.1513/pats.2306032 16113425 10.1513/pats.2306032

[6] McCullagh BN, Comellas AP, Ballas ZK, Newell JD Jr., Zimmerman MB, Azar AE. (2017) Antibody deficiency in patients with frequent exacerbations of Chronic Obstructive Pulmonary Disease (COPD). 12: e0172437. 10.1371/journal.pone.0172437 PMC5315316 28212436

[7] Couper D, LaVange LM, Han M, Barr RG, Bleecker E, Hoffman EA, et al (2014) Design of the Subpopulations and Intermediate Outcomes in COPD Study (SPIROMICS). Thorax 69: 491–494. 10.1136/thoraxjnl-2013-203897 PMC3954445 24029743

[8] O'Neal WK, Anderson W, Basta PV, Carretta EE, Doerschuk CM, Barr RG et al (2014) Comparison of serum, EDTA plasma and P100 plasma for luminex-based biomarker multiplex assays in patients with chronic obstructive pulmonary disease in the SPIROMICS study. J Transl Med 12: 9 doi: 10.1186/1479-5876-12-9 24397870 10.1186/1479-5876-12-9 PMC3928911

[9] Weber-Mzell D, Kotanko P, Hauer AC, Goriup U, Haas J, Lanner N et al (2004) Gender, age and seasonal effects on IgA deficiency: a study of 7293 Caucasians. Eur J Clin Invest 34: 224–228. doi: 10.1111/j.1365-2362.2004.01311.x 15025682 10.1111/j.1365-2362.2004.01311.x

[10] Keene JD, Jacobson S, Kechris K, Kinney GL, Foreman MG, Doerschuk CM et al (2017) Biomarkers Predictive of Exacerbations in the SPIROMICS and COPDGene Cohorts. Am J Respir Crit Care Med 195: 473–481. doi: 10.1164/rccm.201607-1330OC 27579823 10.1164/rccm.201607-1330OC PMC5378424

[11] McCormack MC, Belli AJ, Kaji DA, Matsui EC, Brigham EP, Peng RD et al (2015) Obesity as a susceptibility factor to indoor particulate matter health effects in COPD. Eur Respir J 45: 1248–1257. doi: 10.1183/09031936.00081414 25573407 10.1183/09031936.00081414 PMC4888805

[12] Gonzalez-Quintela A, Alende R, Gude F, Campos J, Rey J, Meijide LM et al (2008) Serum levels of immunoglobulins (IgG, IgA, IgM) in a general adult population and their relationship with alcohol consumption, smoking and common metabolic abnormalities. Clin Exp Immunol 151: 42–50. doi: 10.1111/j.1365-2249.2007.03545.x 18005364 10.1111/j.1365-2249.2007.03545.x PMC2276914

[13] Papi A, Luppi F, Franco F, Fabbri LM (2006) Pathophysiology of exacerbations of chronic obstructive pulmonary disease. Proc Am Thorac Soc 3: 245–251. doi: 10.1513/pats.200512-125SF 16636093 10.1513/pats.200512-125SF

[14] Leitao FRS; Mattman A; Criner G; Woodruff PG; Albert R; Connett JE; et al (2016) Serum Immunoglobulins and Risk of Exacerbations in COPD. Am J Respir Crit Care Med 193: A10108.

[15] Cowan J, Gaudet L, Mulpuru S, Corrales-Medina V, Hawken S, Cameron C et al (2015) A Retrospective Longitudinal Within-Subject Risk Interval Analysis of Immunoglobulin Treatment for Recurrent Acute Exacerbation of Chronic Obstructive Pulmonary Disease. PLoS One 10: e0142205 doi: 10.1371/journal.pone.0142205 26558756 10.1371/journal.pone.0142205 PMC4641695

[16] Webb DR, Condemi JJ (1974) Selective immunoglobulin A deficiency and chronic obstructive lung disease. A family study. Ann Intern Med 80: 618–621. 4545089 10.7326/0003-4819-80-5-618

[17] Richmond BW, Brucker RM, Han W, Du RH, Zhang Y, Cheng DS et al (2016) Airway bacteria drive a progressive COPD-like phenotype in mice with polymeric immunoglobulin receptor deficiency. Nat Commun 7: 11240 doi: 10.1038/ncomms11240 27046438 10.1038/ncomms11240 PMC4822073

[18] Polosukhin VV, Cates JM, Lawson WE, Zaynagetdinov R, Milstone AP, Massion PP et al (2011) Bronchial secretory immunoglobulin a deficiency correlates with airway inflammation and progression of chronic obstructive pulmonary disease. Am J Respir Crit Care Med 184: 317–327. doi: 10.1164/rccm.201010-1629OC 21512171 10.1164/rccm.201010-1629OC PMC3265275

[19] Polosukhin VV, Richmond BW, Du RH, Cates JM, Wu P, Nian H et al (2017) Secretory IgA Deficiency in Individual Small Airways Is Associated with Persistent Inflammation and Remodeling. Am J Respir Crit Care Med 195: 1010–1021. doi: 10.1164/rccm.201604-0759OC 27911098 10.1164/rccm.201604-0759OC PMC5422646

[20] Safaeian M, Kemp T, Falk RT, Rodriguez AC, Hildesheim A, Williams M et al (2009) Determinants and correlation of systemic and cervical concentrations of total IgA and IgG. Cancer Epidemiol Biomarkers Prev 18: 2672–2676. doi: 10.1158/1055-9965.EPI-09-0348 19815637 10.1158/1055-9965.EPI-09-0348 PMC2762345

