# Supplementary material for: Lower serum IgA is associated with COPD exacerbation risk in SPIROMICS
Source: PLoS One. 2018 Apr 12;13(4):e0194924. doi: 10.1371/journal.pone.0194924 (PMC5896903; doi:10.1371/journal.pone.0194924)
Supplement: S1 Table — (DOCX) [file pone.0194924.s001.docx]

**Online only Supporting Information**

**Title:**

**Lower serum IgA is associated with COPD exacerbation risk in SPIROMICS**

**Short Title:**

**Serum IgA and COPD exacerbation risk**

Authors:

Nirupama Putcha*^+1^, Gabriel G. Paul^+1^, Antoine Azar^1^, Robert A. Wise^1^, Wanda K. O’Neal^2^, Mark T. Dransfield^3^, Prescott G. Woodruff^4^, Jeffrey L. Curtis^5,6^, Alejandro P. Comellas^7^, M. Bradley Drummond^2^, Allison A. Lambert^1,14^, Laura M. Paulin^1^, Ashraf Fawzy^1^, Richard E. Kanner^8^, Robert Paine III^9,10^, MeiLan K. Han^5^, Fernando J. Martinez^11^, Russell P. Bowler^12^, R. Graham Barr^13^, Nadia N. Hansel^1^, for the SPIROMICS investigators^#^.

Author Affiliations:

^1^Johns Hopkins University School of Medicine, Baltimore, Maryland, USA

^2^University of North Carolina School of Medicine, Chapel Hill, North Carolina, USA

^3^University of Alabama at Birmingham, Birmingham, Alabama, USA

^4^University of San Francisco School of Medicine, San Francisco, California, USA

^5^University of Michigan Medical School, Ann Arbor, Michigan, USA

^6^VA Ann Arbor Healthcare System, Ann Arbor, Michigan, USA

^7^ University of Iowa College of Medicine, Iowa City, Iowa, USA

^8^University of Utah Health Sciences Center, Salt Lake City, Utah, USA

^9^University of Utah School of Medicine

^10^Department of Veterans Affairs Medical Center, Salt Lake City, Utah, USA

^11^Weill Cornell Medical College. New York City, New York, USA

^12^National Jewish Health, Denver, Colorado, USA

^13^Columbia University School of Medicine, New York, New York, USA

^14^University of Washington School of Medicine, Seattle, Washington, USA

* Corresponding author

Email: [nputcha1@jhmi.edu](mailto:nputcha1@jhmi.edu)

^+^ These authors contributed equally as first author.

^#^Full list of SPIROMICS Investigators can be found in the Acknowledgments section.

**S1 Table: Comparison of characteristics of individuals with IgA level less than or equal to 120 compared to those with IgA level greater than 120.**

| **Characteristics** | **IgA<120 (n=103)** | **IgA>120 (n=945)** | **P-value** |
| --- | --- | --- | --- |
| **IgA level** | 86.2 (23.9) | 289.2 (145.4) |  |
| **Age** | 66.6 (8.92) | 66.0 (7.70) | 0.46 |
| **Female, n(%)** | 48 (47%) | 386 (41%) | 0.26 |
| **African American, n(%)** | 8 (8%) | 126 (13%) | 0.11 |
| **BMI** | 27.1.0 (4.83) | 27.5 (5.26) | 0.38 |
| **> HS education, n(%)** | 66 (64%) | 596 (63%) | 0.86 |
| **Follow-up time (days)** | 891.8 (312.5) | 949.9 (277.2) | 0.05 |
| **Smoking History (Pack-yrs)** | 48.7 (20.6) | 55.2 (27.1) | 0.02 |
| **Current Smokers, n(%)** | 42 (41%) | 296 (31%) | 0.05 |
| **Post-FEV1% Predicted** | 63.9 (23.4) | 62.2 (23.1) | 0.47 |
| **Gold Stage n(%)**  **1**  **2**  **3**  **4** | 29 (28%)  44 (43%)  23 (22%)  7 (7%) | 228 (24%)  416 (44%)  218 (23%)  83 (9%) | 0.77 |
| **MMRC Dyspnea Score** | 1.24 (0.95) | 1.21 (1.02) | 0.82 |
| **Current oral steroid use, n(%)** | 1 (4%) | 33 (3%) | 0.81 |
| **SGRQ Score** | 36.5 (1.75) | 36.0 (19.6) | 0.80 |
| **>2 exacerbations over follow-up, n(%)** | 31 (30%) | 290 (31%) | 0.84 |

***All values mean (SD) unless otherwise indicated.**
